# Supplementary figures and images for: Job satisfaction among healthcare workers in the aftermath of the COVID-19 pandemic
Source: PLoS One. 2022 Oct 26;17(10):e0275334. doi: 10.1371/journal.pone.0275334 (PMC9603954; doi:10.1371/journal.pone.0275334)

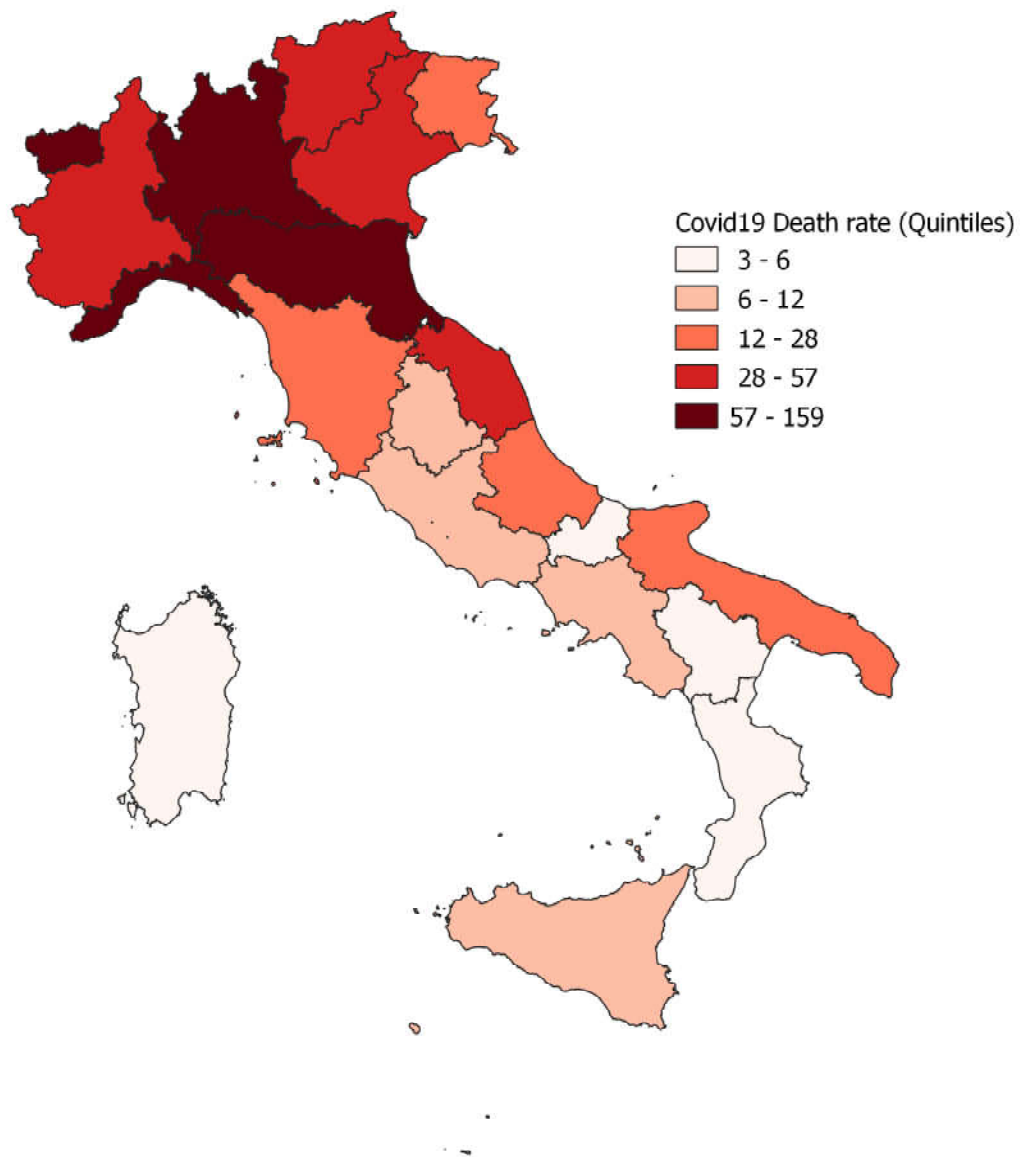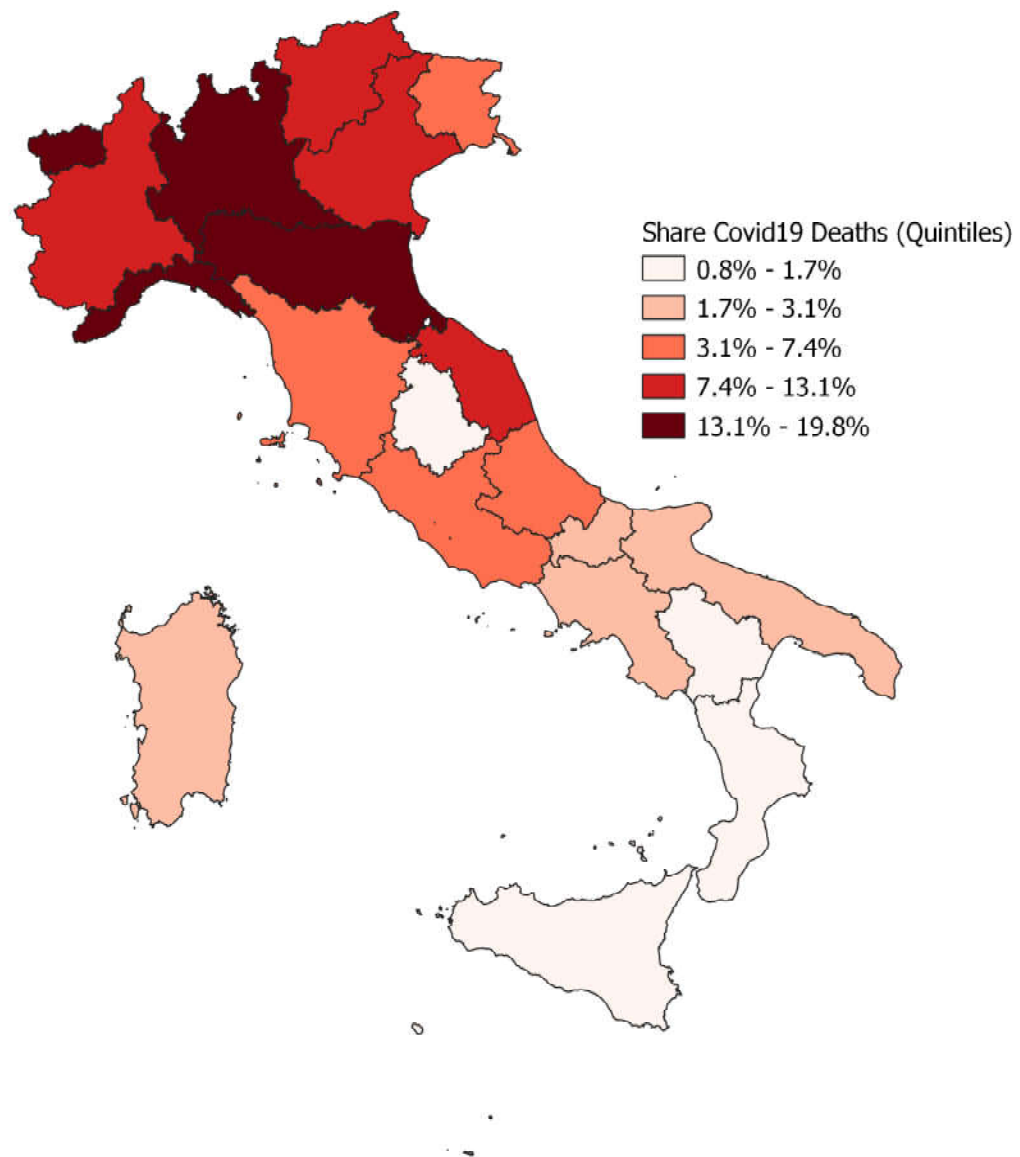

Supplement: S1 Fig — The Death Rates (A) and Share of COVID-19 Deaths (B) are measures computed by the National Institute of Statistics (Istat) together with the Istituto Superiore di Sanitá (Iss) on administrative data [33]. The index Death Rates, referring to the period January-May 2020, represents the mortality rate due to COVID-19 standardized by the demographic characteristics of the resident population in each province (values expressed per 100,000 inhabitants). The Share of COVID-19 Deaths is the proportion of deaths by COVID-19 cases over the total number of deaths in the relevant time and location. The shapefile used to create the map is plotting the boundaries of the regional administrative units as of January 2020 and it is retrieved from the National Institute of Statistics (Istat). Elaboration by the authors. (PDF) [file pone.0275334.s001.pdf]

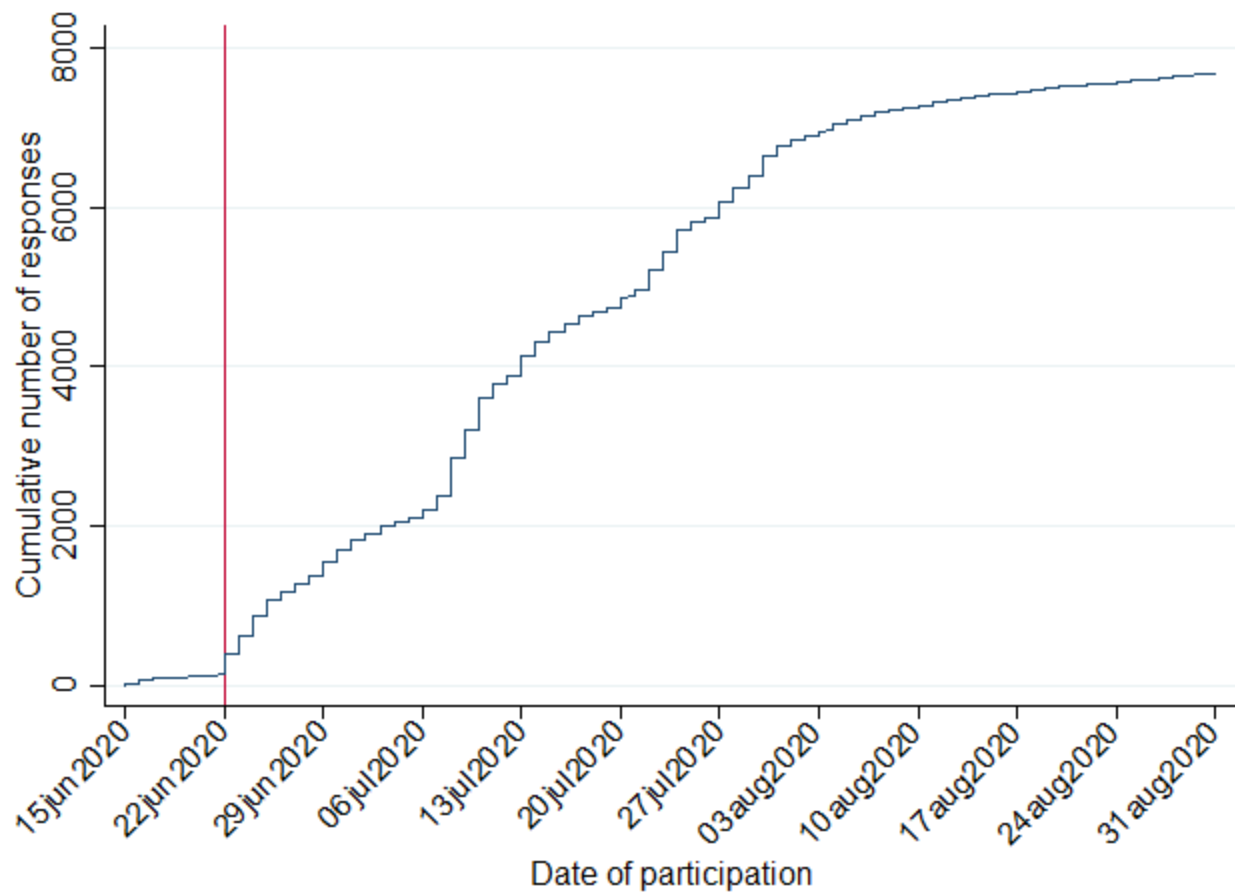

Supplement: S2 Fig — The graph shows the cumulative number of responses to the online survey by day of participation (i.e., day when the completed survey was submitted and the response was registered by the Google Form platform). The vertical line identifies the end of the pilot run during the first week (June 15th to June 22, 2020) to verify the clarity of the questionnaire. No issue arose during the pilot, and therefore we proceeded using all responses collected in the analysis. (PDF) [file pone.0275334.s002.pdf]

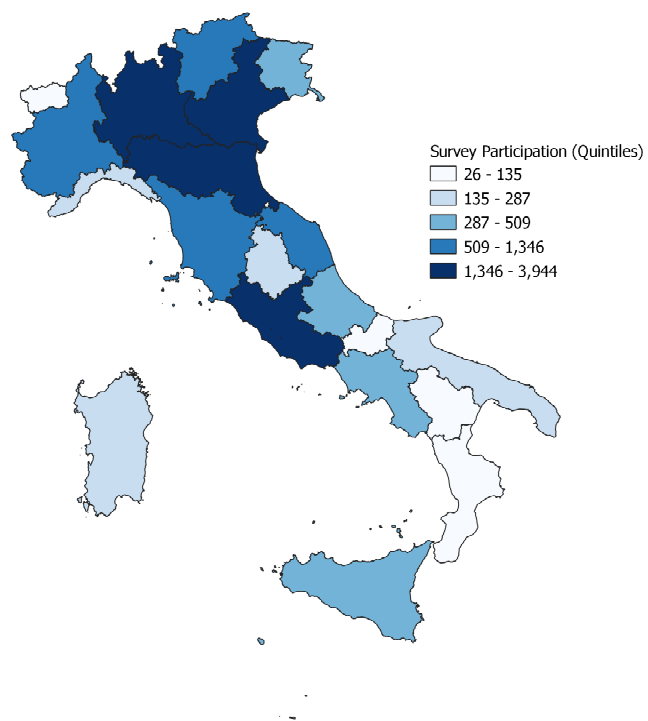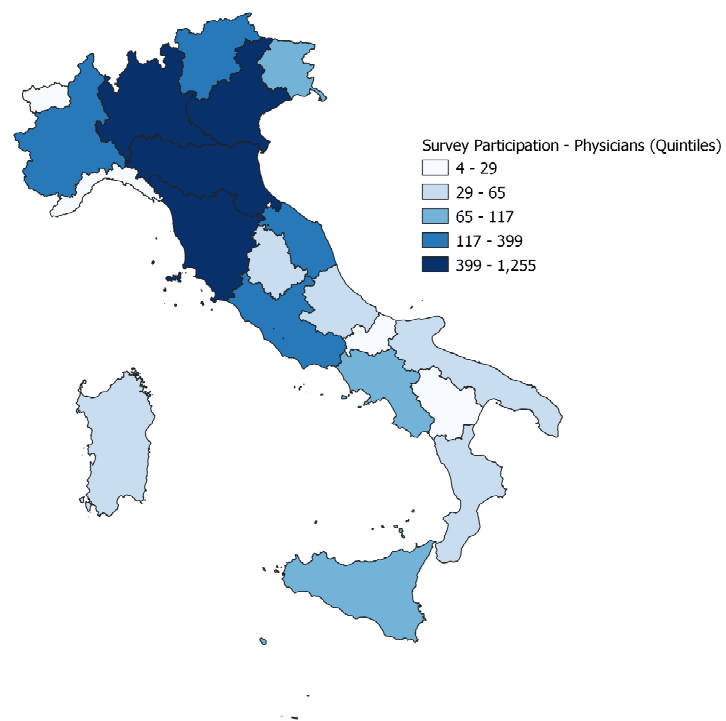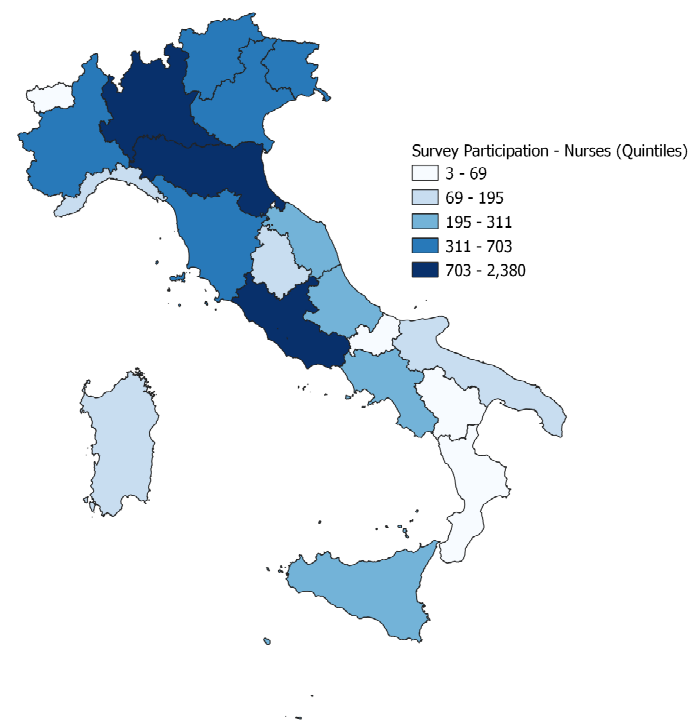

Supplement: S4 Fig — The maps present the absolute numbers of participants by region and professional category, i.e., all healthcare workers (A), physicians (B), and nurses (C). Data reported by quintiles of the professional category considered. The shapefile used to create the map is plotting the boundaries of the regional administrative units as of January 2020 and it is retrieved from the National Institute of Statistics (Istat). Elaboration by the authors. (PDF) [file pone.0275334.s004.pdf]
