# Supplementary material for: Job satisfaction among healthcare workers in the aftermath of the COVID-19 pandemic
Source: PLoS One. 2022 Oct 26;17(10):e0275334. doi: 10.1371/journal.pone.0275334 (PMC9603954; doi:10.1371/journal.pone.0275334)
Supplement: S3 Fig — (PDF) [file pone.0275334.s003.pdf]

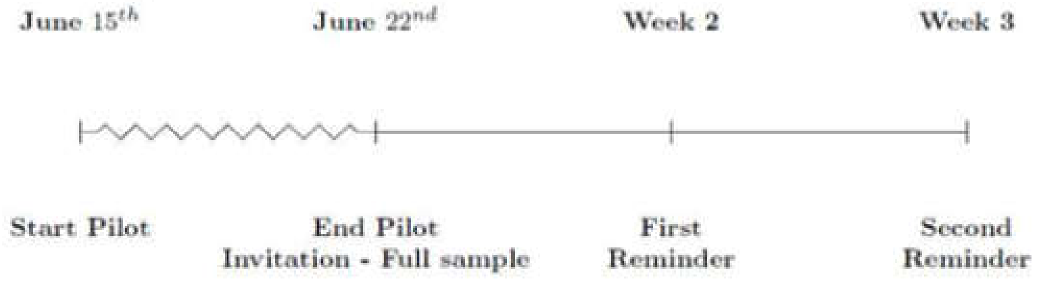
June 15th
June 22nd
Week 2
Week 3
Start Pilot
End Pilot
Invitation - Full sample
First
Reminder
Second
Reminder
