# Supplementary material for: Job satisfaction among healthcare workers in the aftermath of the COVID-19 pandemic
Source: PLoS One. 2022 Oct 26;17(10):e0275334. doi: 10.1371/journal.pone.0275334 (PMC9603954; doi:10.1371/journal.pone.0275334)
Supplement: S1 Table — Professional associations that shared our survey with their members through their website and/or their mailing list. (PDF) [file pone.0275334.s005.pdf]

S1 Table.

S1 Table. Associations Participating in the Survey

Description	Professionals
Segretariato Italiano Giovani Medici	Physicians
Associazione Anestesisti Rianimatori Ospedalieri Italiani - Emergenza Area Critica	Physicians
Organizzazione Sindacale interdisciplinare e apartitica dei Medici Ospedalieri Dipendenti dal S.S.N.	Physicians
Associazione Italiana Nursing Sociale	Nurses
Associazione Infermieri di Famiglia e di Comunità	Nurses

Professional associations that shared our survey with their members through their website and/or their mailing list.
