# Supplementary material for: Job satisfaction among healthcare workers in the aftermath of the COVID-19 pandemic
Source: PLoS One. 2022 Oct 26;17(10):e0275334. doi: 10.1371/journal.pone.0275334 (PMC9603954; doi:10.1371/journal.pone.0275334)
Supplement: S2 Table — Definition of the outcomes of interest, alternative outcomes and individual components of satisfaction. Individual components are defined as dummies, moving from categorical variables originally measured on a 5-item scale. (PDF) [file pone.0275334.s006.pdf]

S2 Table.

S2 Table. Outcomes Definition

Outcomes	Definition
**Outcomes of interest**	
Satisfaction	Discrete from 0 to 8, with 8 being the highest value. It is the sum of 8 dummies: Profession, Work, Salary, Work-Life balance, Relationship with colleagues and administration, Hours of work, Career path.
Profession change	Dummy=1 for medium to high agreement with the statement "If I could start over, I would not be in this profession"
Specialization change	Dummy=1 for medium to high agreement with the statement "If I could start over, I would choose a different field of specialization"
**Alternative outcomes**	
Satisfaction 2	Discrete from 8 to 40, with 40 being the highest value. It is the sum of 8 categorical variables (5-items scale): Profession, Work, Salary, Work-Life balance, Relationship with colleagues and administration, Hours of work, Career path.
Satisfaction 3	Discrete from 1 to 5, with 5 being the highest value. It is the arithmetic mean of 8 categorical variables (5-items scale) : Profession, Work, Salary, Work-Life balance, Relationship with colleagues and administration, Hours of work, Career path.
Satisfaction PCA	Continuous measure ranging from -5.42 to 4.69 (mean 0). It is the first principal component derived from a PCA analysis on 8 categorical variables (5-item scale): Profession, Work, Salary, Work-Life balance, Relationship with colleagues and administration, Hours of work, Career path.
**Individual components**	
Profession	Dummy=1 if the worker is satisfied with the profession: extremely or averagely and 0 otherwise
Work	Dummy=1 if the worker is satisfied with the current job: extremely or averagely and 0 otherwise
Salary	Dummy=1 if the worker is satisfied with the current pay: extremely or averagely and 0 otherwise
Work-Life balance	Dummy=1 if the worker is satisfied with the balance between private and work commitments: extremely or averagely and 0 otherwise
Relationship with colleagues	Dummy=1 if the worker is satisfied with the relationship with the colleagues: extremely or averagely and 0 otherwise
Relationship with administration	Dummy=1 if the worker is satisfied with the relationship with the administration: extremely or averagely and 0 otherwise
Hours of work	Dummy=1 if the worker is satisfied with the number of hours worked: extremely or averagely and 0 otherwise
Career Path	Dummy=1 if the worker is satisfied with the career prospects: extremely or averagely and 0 otherwise

Definition of the outcomes of interest, alternative outcomes and individual components of satisfactions. Individual components are defined as dummies, moving from categorical variables originally measures on a 5-items scale.
