# Supplementary material for: Job satisfaction among healthcare workers in the aftermath of the COVID-19 pandemic
Source: PLoS One. 2022 Oct 26;17(10):e0275334. doi: 10.1371/journal.pone.0275334 (PMC9603954; doi:10.1371/journal.pone.0275334)
Supplement: S3 Table — When we refer to the COVID-19 crisis, we refer to the first wave that took place in Italy from the end of February 2020 to the beginning of June 2020. AMI = Acute myocardial infraction. COPD = Chronic obstructive pulmonary disease. All 30-day readmission rates have been collected from the ‘National Healthcare Outcomes Program” (“Piano Nazionale Esiti—PNE), which is a national program run since 2012 by the Ministry of Health intended to develop and implement practical indexes to measure, analyze, evaluate and monitor the performance of healthcare facilities operating within the Italian healthcare system. (PDF) [file pone.0275334.s007.pdf]

S3 Table.

S3 Table. Variables Definition

Variable	Definition
Children	Dummy=1 if you have children and 0 otherwise
Age	Categorical variable: <30, 30-40, 40-50, 50-60, >60.
Female	Dummy=1 if you are female and 0 otherwise
Italian	Dummy=1 if you are Italian and 0 otherwise
Married	Dummy=1 if you are married and 0 otherwise
House sq. meter > 100	Dummy=1 if your home size is bigger than 100 squared meters and 0 otherwise
Good health status	Dummy=1 if you classify your health status as good or very good and 0 otherwise
Chronic disease	Dummy=1 if you have at least one chronic disease
Living alone	Dummy=1 if you live alone and 0 otherwise
Never changed workplace	Dummy=1 if you have always worked in the same workplace and 0 otherwise
Health workers in the family	Dummy=1 if in your family of origin there are healthcare workers and 0 otherwise
Hospital worker	Dummy=1 if you work in a hospital and 0 otherwise
Teaching hospital	Dummy=1 if you work in a teaching hospital and 0 otherwise
Private	Dummy=1 if you work for the private sector and 0 otherwise
Managerial role	Dummy=1 for workers with managerial or coordinating roles
Contract with work-shifts	Dummy=1 if you have a contract which requires work-shifts and 0 otherwise
Average hours worked	Continuous variable indicating the average number of hours worked per week.
Tenure	Continuous variable indicating the number of years of work.
COVID-19 specialization	Dummy=1 for "ICU", "Pneumology", "Infectious Diseases", "Anaesthesia" and "Emergency Room" wards.
High quality facility	Dummy=1 for workplaces with very good or higher quality.
Lack of medical personnel	Dummy=1 for a medium to high lack of the medical personnel in the province of work.
High salary	Dummy=1 for workers earning at least 2,000€net per month.
COVID-19 Death rate	Continuous variable measuring the adjusted COVID-19 death rate over 100,000 inhabitants at the provincial level.
Prompt response	Dummy=1 for good or higher promptness of the new regulation.
Effective response	Dummy=1 for good or higher effectiveness of the new regulation.
Infected colleagues	Dummy=1 for one or more infected or hospitalized colleagues.
Dead colleagues	Dummy=1 for one or more dead colleagues.
COVID-19 overtime	Dummy=1 for COVID-19 related overtime at work.
Exposed to COVID-19	Dummy=1 for workers quarantined as suspicious contacts.
Positive to COVID-19	Dummy=1 for quarantined workers with symptoms.
Work with COVID-19 positives	Dummy=1 for working with COVID-19 positives.
COVID-19: change of specialization/function	Dummy=1 for a change in specialization/function due to COVID-19.
30 days readmission rate for stroke	Continuous variables computed as the number of readmission due to stroke within 30 days from discharge out the total number of admissions due to stroke
30 days readmission rate for AMI	Continuous variables computed as the number of readmission due to AMI within 30 days from discharge out the total number of admissions due to AMI
30 days readmission rate for COPD	Continuous variables computed as the number of readmission due to COPD within 30 days from discharge out the total number of admissions due to COPD

When we refer to the COVID-19 crisis, we refer to the first wave that took place in Italy from the end of February 2020 to the beginning of June 2020. AMI=Acute myocardial infraction. COPD=Chronic obstructive pulmonary disease. All 30days readmission rates have been collected from the 'National Healthcare Outcomes Program" ("Piano Nazionale Esiti - PNE) which is a national program run since 2012 by the Ministry of Health aiming at developing and implementing practical indexes to measure, analyze, evaluate and monitor the performances of healthcare facilities operating within the Italian healthcare system.
