# Supplementary material for: Job satisfaction among healthcare workers in the aftermath of the COVID-19 pandemic
Source: PLoS One. 2022 Oct 26;17(10):e0275334. doi: 10.1371/journal.pone.0275334 (PMC9603954; doi:10.1371/journal.pone.0275334)
Supplement: S4 Table — OLS regressions. See S2 and S3 Tables for outcome and control definitions, respectively. Standard errors clustered at the level of the region of work in parentheses. Significant at 10% *; significant at 5% **; significant at 1% ***. (PDF) [file pone.0275334.s008.pdf]

S4 Table.

S4 Table. Satisfaction and Willingness to Change Profession or Specialization - Robustness on COVID-19 Factors

	Satisfaction			Profession change			Specialization change		
	(1)	(2)	(3)	(4)	(5)	(6)	(7)	(8)	(9)
**Personal factors:**									
Children	0.018	-0.012	0.014	0.018	-0.012	0.014	0.018	-0.012	0.014
	(0.060)	(0.019)	(0.013)	(0.060)	(0.019)	(0.013)	(0.060)	(0.019)	(0.013)
Age: >=30 - <40	-0.610***	0.091***	0.093***	-0.610***	0.091***	0.093***	-0.610***	0.091***	0.093***
	(0.078)	(0.024)	(0.016)	(0.078)	(0.024)	(0.016)	(0.078)	(0.024)	(0.016)
Age: >=40 - <50	-0.674***	0.115***	0.106***	-0.674***	0.115***	0.106***	-0.674***	0.115***	0.106***
	(0.089)	(0.036)	(0.018)	(0.089)	(0.036)	(0.018)	(0.089)	(0.036)	(0.018)
Age: >=50 - <60	-0.583***	0.062*	0.094***	-0.583***	0.062*	0.094***	-0.583***	0.062*	0.094***
	(0.103)	(0.034)	(0.017)	(0.103)	(0.034)	(0.017)	(0.103)	(0.034)	(0.017)
Age: >=60	-0.404**	-0.004	0.060**	-0.404**	-0.004	0.060**	-0.404**	-0.004	0.060**
	(0.142)	(0.043)	(0.027)	(0.142)	(0.043)	(0.027)	(0.142)	(0.043)	(0.027)
Female	-0.064	-0.047***	-0.049**	-0.064	-0.047***	-0.049**	-0.064	-0.047***	-0.049**
	(0.047)	(0.012)	(0.018)	(0.047)	(0.012)	(0.018)	(0.047)	(0.012)	(0.018)
Italian	0.074	-0.005	-0.105**	0.074	-0.005	-0.105**	0.074	-0.005	-0.105**
	(0.191)	(0.044)	(0.042)	(0.191)	(0.044)	(0.042)	(0.191)	(0.044)	(0.042)
Married	0.150**	-0.002	-0.012	0.150**	-0.002	-0.012	0.150**	-0.002	-0.012
	(0.062)	(0.014)	(0.016)	(0.062)	(0.014)	(0.016)	(0.062)	(0.014)	(0.016)
House sq. meters >100	0.044	-0.026**	-0.016	0.044	-0.026**	-0.016	0.044	-0.026**	-0.016
	(0.035)	(0.011)	(0.012)	(0.035)	(0.011)	(0.012)	(0.035)	(0.011)	(0.012)
Good health status	0.918***	-0.109***	-0.112***	0.918***	-0.109***	-0.112***	0.918***	-0.109***	-0.112***
	(0.122)	(0.026)	(0.035)	(0.122)	(0.026)	(0.035)	(0.122)	(0.026)	(0.035)
Chronic diseases	-0.330***	0.061***	0.041**	-0.330***	0.061***	0.041**	-0.330***	0.061***	0.041**
	(0.051)	(0.014)	(0.016)	(0.051)	(0.014)	(0.016)	(0.051)	(0.014)	(0.016)
Living alone	0.051	-0.003	-0.036	0.051	-0.003	-0.036	0.051	-0.003	-0.036
	(0.078)	(0.025)	(0.023)	(0.078)	(0.025)	(0.023)	(0.078)	(0.025)	(0.023)
Never changed workplace	0.108	-0.025***	-0.039***	0.108	-0.025***	-0.039***	0.108	-0.025***	-0.039***
	(0.071)	(0.008)	(0.013)	(0.071)	(0.008)	(0.013)	(0.071)	(0.008)	(0.013)
Health workers in the family	0.026	0.015	0.010	0.026	0.015	0.010	0.026	0.015	0.010
	(0.051)	(0.009)	(0.012)	(0.051)	(0.009)	(0.012)	(0.051)	(0.009)	(0.012)
**Contextual factors:**									
Hospital worker	-0.275***	0.026	-0.004	-0.275***	0.026	-0.004	-0.275***	0.026	-0.004
	(0.051)	(0.018)	(0.013)	(0.051)	(0.018)	(0.013)	(0.051)	(0.018)	(0.013)
Teaching hospital	-0.208	0.011	-0.060***	-0.208	0.011	-0.060***	-0.208	0.011	-0.060***
	(0.148)	(0.029)	(0.020)	(0.148)	(0.029)	(0.020)	(0.148)	(0.029)	(0.020)
Private	-0.088	0.004	-0.014	-0.088	0.004	-0.014	-0.088	0.004	-0.014
	(0.107)	(0.015)	(0.016)	(0.107)	(0.015)	(0.016)	(0.107)	(0.015)	(0.016)
Management role	0.233***	-0.058***	-0.024	0.233***	-0.058***	-0.024	0.233***	-0.058***	-0.024
	(0.057)	(0.019)	(0.018)	(0.057)	(0.019)	(0.018)	(0.057)	(0.019)	(0.018)
Contract with work-shifts	-0.441***	-0.000	0.023	-0.441***	-0.000	0.023	-0.441***	-0.000	0.023
	(0.049)	(0.027)	(0.018)	(0.049)	(0.027)	(0.018)	(0.049)	(0.027)	(0.018)
Average hours worked	-0.042***	0.003***	0.003***	-0.042***	0.003***	0.003***	-0.042***	0.003***	0.003***
	(0.004)	(0.001)	(0.001)	(0.004)	(0.001)	(0.001)	(0.004)	(0.001)	(0.001)
Tenure	-0.003	0.001	-0.000	-0.003	0.001	-0.000	-0.003	0.001	-0.000
	(0.004)	(0.001)	(0.001)	(0.004)	(0.001)	(0.001)	(0.004)	(0.001)	(0.001)
COVID-19 specialization	0.022	0.009	-0.021*	0.022	0.009	-0.021*	0.022	0.009	-0.021*
	(0.079)	(0.013)	(0.011)	(0.079)	(0.013)	(0.011)	(0.079)	(0.013)	(0.011)
High quality facility	1.058***	-0.095***	-0.113***	1.058***	-0.095***	-0.113***	1.058***	-0.095***	-0.113***
	(0.030)	(0.019)	(0.015)	(0.030)	(0.019)	(0.015)	(0.030)	(0.019)	(0.015)
Lack of medical personnel	-0.228***	0.038**	0.048**	-0.228***	0.038**	0.048**	-0.228***	0.038**	0.048**
	(0.045)	(0.014)	(0.019)	(0.045)	(0.014)	(0.019)	(0.045)	(0.014)	(0.019)
High salary	0.640***	-0.061***	-0.068***	0.640***	-0.061***	-0.068***	0.640***	-0.061***	-0.068***
	(0.081)	(0.016)	(0.023)	(0.081)	(0.016)	(0.023)	(0.081)	(0.016)	(0.023)
Nurse	0.008	0.037	0.018	0.008	0.037	0.018	0.008	0.037	0.018
	(0.110)	(0.025)	(0.029)	(0.110)	(0.025)	(0.029)	(0.110)	(0.025)	(0.029)
**COVID-19 related factors:**									
COVID-19 Death rate	0.001***		-0.000**	0.001***		-0.000**	0.001***		-0.000**
	(0.000)		(0.000)	(0.000)		(0.000)	(0.000)		(0.000)
Prompt response		-0.009	0.002		-0.009	0.002		-0.009	0.002
		(0.009)	(0.012)		(0.009)	(0.012)		(0.009)	(0.012)
Effective response		-0.034*	-0.038***		-0.034*	-0.038***		-0.034*	-0.038***
		(0.017)	(0.010)		(0.017)	(0.010)		(0.017)	(0.010)
Infected colleagues		0.010	0.009		0.010	0.009		0.010	0.009
		(0.019)	(0.008)		(0.019)	(0.008)		(0.019)	(0.008)
Dead colleagues		0.024	-0.022		0.024	-0.022		0.024	-0.022
		(0.022)	(0.026)		(0.022)	(0.026)		(0.022)	(0.026)
COVID-19 overtime		0.006	0.008		0.006	0.008		0.006	0.008
		(0.011)	(0.013)		(0.011)	(0.013)		(0.011)	(0.013)
Exposed to COVID19		0.007	-0.003		0.007	-0.003		0.007	-0.003
		(0.020)	(0.022)		(0.020)	(0.022)		(0.020)	(0.022)
Positive to COVID19		-0.016	0.005		-0.016	0.005		-0.016	0.005
		(0.017)	(0.019)		(0.017)	(0.019)		(0.017)	(0.019)
Work with COVID19 positives		-0.036***	-0.040***		-0.036***	-0.040***		-0.036***	-0.040***
		(0.009)	(0.009)		(0.009)	(0.009)		(0.009)	(0.009)
COVID-19: change of specialization/function		0.034*	0.033**		0.034*	0.033**		0.034*	0.033**
		(0.018)	(0.015)		(0.018)	(0.015)		(0.018)	(0.015)
Constant	6.716***	0.407***	0.476***	6.716***	0.407***	0.476***	6.716***	0.407***	0.476***
	(0.334)	(0.075)	(0.068)	(0.334)	(0.075)	(0.068)	(0.334)	(0.075)	(0.068)
N Obs.	7,134	7,134	7,134	7,134	7,134	7,134	7,134	7,134	7,134
Macro area fixed effect	No	No	No	No	No	No	No	No	No
Region fixed effect	Yes	Yes	Yes	Yes	Yes	Yes	Yes	Yes	Yes
Clustered standard errors	Yes	Yes	Yes	Yes	Yes	Yes	Yes	Yes	Yes

OLS regressions. See S2 Table and S3 Table for outcomes and controls definitions, respectively. Standard errors clustered at the level of the region of work in parentheses. Significant at 10% *; significant at 5% **; significant at 1% ***.
