# Supplementary material for: Job satisfaction among healthcare workers in the aftermath of the COVID-19 pandemic
Source: PLoS One. 2022 Oct 26;17(10):e0275334. doi: 10.1371/journal.pone.0275334 (PMC9603954; doi:10.1371/journal.pone.0275334)
Supplement: Attachment — Submitted filename: respone_reviewers.pdf [file pone.0275334.s011.pdf]

We thank the Editor for the comments/guidelines he gave us. In what follows, we answer to each of the specific points raised by him detailing how these have been taken into account in the revised version.

COMMENTS AND RELATED REPLIES

When submitting your revision, we need you to address these additional requirements.

1. Please ensure that your manuscript meets PLOS ONE's style requirements, including those for file naming. The PLOS ONE style templates can be found at https://journals.plos.org/plosone/s/file?id=wjVg/PLOSOne_formatting_sample_main_body.pdf and https://journals.plos.org/plosone/s/file?id=ba62/PLOSOne_formatting_sample_title_authors_affiliations.pdf

A: The manuscript has been reviewed to ensure compliance with PLOS ONE's style requirements. Specifically, we adapt the Title formatting and insert the correct authors' information. We eliminate the Section and Subsection numbering. The main sections are now Introduction, Materials and Methods, Results and Discussion, Conclusion. We took care of Tables and Figures formatting the related captions, as well as of footnotes.

2. Please provide additional details regarding participant consent. In the ethics statement in the Methods and online submission information, please ensure that you have specified (1) whether consent was informed and (2) what type you obtained (for instance, written or verbal, and if verbal, how it was documented and witnessed). If your study included minors, state whether you obtained consent from parents or guardians. If the need for consent was waived by the ethics committee, please include this information.

If you are reporting a retrospective study of medical records or archived samples, please ensure that you have discussed whether all data were fully anonymized before you accessed them and/or whether the IRB or ethics committee waived the requirement for informed consent. If patients provided informed written consent to have data from their medical records used in research, please include this information.

A: We obtained written informed consent from each participant at the beginning of the questionnaire. Specifically, the first screen provided participants with information about the content of the survey, its intended use and a privacy statement. After this information, each participant was asked whether she agreed to provide her consent for participation. If participants ticked the option ``proceed" after the statement ``I confirm that I have read the information on the processing of personal data and I agree to participate in this survey" on the electronic form, the survey would begin. Respondents voluntarily participated and could withdraw from the survey at any time. Our study does not include minors and does not use medical records or achieved samples. This is now explained in the Section "Ethics".

3. During the internal evaluation of the submission we have noted the following statement: "this

research was approved by the Ethics committee of the University of Pavia (Italy) to which Emilia Barili affiliated at the time of the survey" Please could you clarify whether author Emilia Barili is affiliated with the research ethics committee which provided ethical approval. If no, please could you revise this text to avoid confusion.

A: Emilia Barili was affiliated with the University of Pavia at the time of the survey, but she was not directly affiliated or connected to the Ethics committee of the University of Pavia

4. Please update your submission to use the PLOS LaTeX template. The template and more information on our requirements for LaTeX submissions can be found at http://journals.plos.org/plosone/s/latex.

A: We adopted the PLOS LaTex template as requested.

5. Thank you for stating the following financial disclosure:

"Paola Bertoli is the recipient of a Rita Levi Montalcini Fellowship to promote the moving back in Italian University of Young Italian Scholars based abroad and willing to come back to Italy"

Please state what role the funders took in the study. If the funders had no role, please state: "The funders had no role in study design, data collection and analysis, decision to publish, or preparation of the manuscript."
If this statement is not correct you must amend it as needed.
Please include this amended Role of Funder statement in your cover letter; we will change the online submission form on your behalf.

A: The funder had no role in study design, data collection and analysis, decision to publish, or preparation of the manuscript.

6. Please include your full ethics statement in the 'Methods' section of your manuscript file. In your statement, please include the full name of the IRB or ethics committee who approved or waived your study, as well as whether or not you obtained informed written or verbal consent. If consent was waived for your study, please include this information in your statement as well.

A: We now specify in the "Materials and Methods" section under "Ethics" what follows: "The study protocol was approved by the ``Comitato di Approvazione per la Ricerca sull'Uomo'', that is, the Ethics Committee of the University of Verona (Prot. N. 0221872 - 22/06/2020). The protocol was also registered at AEA-RCT registry (AEARCTR-0007419), while the University of Pavia certified compliance with privacy requirements (Prot. N. 61080 - 15/06/2020). All participants gave their written informed consent that was embedded on the first page of the questionnaire. After reading a description of the questionnaire, healthcare professionals were asked if they agreed to participate. If they ticked the option ``proceed" after the statement ``I confirm that I have read the information on the processing of personal data and I agree to participate in this survey'' on the electronic form, the survey would begin. Respondents voluntarily participated and could withdraw from the survey at any time."

7. We note that Figures 1 and 3 in your submission contain copyrighted images. All PLOS content is published under the Creative Commons Attribution License (CC BY 4.0), which means that the manuscript, images, and Supporting Information files will be freely available online, and any third party is permitted to access, download, copy, distribute, and use these materials in any way, even commercially, with proper attribution. For more information, see our copyright guidelines: http://journals.plos.org/plosone/s/licenses-and-copyright.

We require you to either (1) present written permission from the copyright holder to publish these figures specifically under the CC BY 4.0 license, or (2) remove the figures from your submission:

a. You may seek permission from the original copyright holder of Figures 1 and 3 to publish the content specifically under the CC BY 4.0 license.

We recommend that you contact the original copyright holder with the Content Permission Form (http://journals.plos.org/plosone/s/file?id=7c09/content-permission-form.pdf) and the following text:
“I request permission for the open-access journal PLOS ONE to publish XXX under the Creative Commons Attribution License (CCAL) CC BY 4.0 (http://creativecommons.org/licenses/by/4.0/). Please be aware that this license allows unrestricted use and distribution, even commercially, by third parties. Please reply and provide explicit written permission to publish XXX under a CC BY license and complete the attached form.”

Please upload the completed Content Permission Form or other proof of granted permissions as an "Other" file with your submission.

In the figure caption of the copyrighted figure, please include the following text: “Reprinted from [ref] under a CC BY license, with permission from [name of publisher], original copyright [original copyright year].”

b. If you are unable to obtain permission from the original copyright holder to publish these figures under the CC BY 4.0 license or if the copyright holder’s requirements are incompatible with the CC BY 4.0 license, please either i) remove the figure or ii) supply a replacement figure that complies with the CC BY 4.0 license. Please check copyright information on all replacement figures and update the figure caption with source information. If applicable, please specify in the figure caption text when a figure is similar but not identical to the original image and is therefore for illustrative purposes only.

A: Figures 1 and 3 do not contain copyrighted images, but rather images we produced. We now specify this in the footnotes to the figures.

Additional Editor Comments:

Thank you for submitting the results of your study to the PLOS ONE.
Prior to further steps, it is necessary to organize your manuscript per the PLOS ONE's guidelines

(https://journals.plos.org/plosone/s/submission-guidelines):
Manuscripts should be organized as follows:

Beginning section
The following elements are required, in order:

Title page: List title, authors, and affiliations as first page of the manuscript
Abstract
Introduction
Middle section
The following elements can be renamed as needed and presented in any order:

Materials and Methods
Results
Discussion
Conclusions (optional)
Ending section
The following elements are required, in order:

Acknowledgments
References
Supporting information captions (if applicable)
Other elements
Figure captions are inserted immediately after the first paragraph in which the figure is cited.
Figure files are uploaded separately.
Tables are inserted immediately after the first paragraph in which they are cited.
Supporting information files are uploaded separately.

A: We re-organized the structure of the paper as requested. In particular, the paper now includes Abstract, Introduction, a section providing information on the institutional setting, Materials and Methods, Results and Discussion, Conclusions. We also included the applicable required elements as, among others, acknowledgements and references.
