# Supplementary material for: Job satisfaction among healthcare workers in the aftermath of the COVID-19 pandemic
Source: PLoS One. 2022 Oct 26;17(10):e0275334. doi: 10.1371/journal.pone.0275334 (PMC9603954; doi:10.1371/journal.pone.0275334)
Supplement: Attachment — Submitted filename: reply_reviewers.pdf [file pone.0275334.s012.pdf]

Dear Prof. Taghizadeh-Hesary,

I am writing regarding your letter about the manuscript PONE-D-22-00138 titled "Job Satisfaction Among Healthcare Workers in the Aftermath of the COVID-19 Pandemic".

I would like to thank you, and the four Reviewers for the prompt feedback about our submission, and for the positive comments and the opportunity to revise the manuscript.

We have addressed each of the points raised in the reports and attach a document that contains the Reviewers' comments highlighted in *italics* and our replies right below each comment.

We greatly appreciate the opportunity to improve the manuscript, and we hope that this revised version will bring us nearer to a publication in PLOS ONE.

We are very much looking forward to your response.

Best regards,

Emilia Barili, Paola Bertoli, Veronica Grembi and Veronica Rattini

Reviewers' comments:

Reviewer's Responses to Questions

Comments to the Author

1. If the authors have adequately addressed your comments raised in a previous round of review and you feel that this manuscript is now acceptable for publication, you may indicate that here to bypass the “Comments to the Author” section, enter your conflict of interest statement in the “Confidential to Editor” section, and submit your "Accept" recommendation.

Reviewer #1: All comments have been addressed

Reviewer #2: All comments have been addressed

Reviewer #3: (No Response)

Reviewer #4: All comments have been addressed

2. Is the manuscript technically sound, and do the data support the conclusions?

The manuscript must describe a technically sound piece of scientific research with data that supports the conclusions. Experiments must have been conducted rigorously, with appropriate controls, replication, and sample sizes. The conclusions must be drawn appropriately based on the data presented.

Reviewer #1: Yes

Reviewer #2: Yes

Reviewer #3: Partly

Reviewer #4: Yes

---

3. Has the statistical analysis been performed appropriately and rigorously?

Reviewer #1: Yes

Reviewer #2: Yes

Reviewer #3: Yes

Reviewer #4: Yes

---

4. Have the authors made all data underlying the findings in their manuscript fully available?

The PLOS Data policy requires authors to make all data underlying the findings described in their manuscript fully available without restriction, with rare exception (please refer to the Data Availability Statement in the manuscript PDF file). The data should be provided as part of the manuscript or its supporting information, or deposited to a public repository. For example, in addition to summary statistics, the data points behind means, medians and variance measures should be available. If there are restrictions on publicly sharing data—e.g. participant privacy or use of data from a third party—those must be specified.

Reviewer #1: Yes

Reviewer #2: Yes

Reviewer #3: Yes

Reviewer #4: Yes

---

5. Is the manuscript presented in an intelligible fashion and written in standard English?

PLOS ONE does not copyedit accepted manuscripts, so the language in submitted articles must be clear, correct, and unambiguous. Any typographical or grammatical errors should be corrected at revision, so please note any specific errors here.

Reviewer #1: Yes

Reviewer #2: Yes

Reviewer #3: No

Reviewer #4: Yes

---

6. Review Comments to the Author

Please use the space provided to explain your answers to the questions above. You may also include additional comments for the author, including concerns about dual publication, research

ethics, or publication ethics. (Please upload your review as an attachment if it exceeds 20,000 characters)

Reviewer #1:

Manuscript presents a current and important theme in the elaboration of human resources policies in the health area, the COVID-19 pandemic was a learning experience, including the need for rapid activation of emergency programs in public health. It presents important and significant results. I add some considerations for the authors' evaluation.

We thank Reviewer 1 for the positive assessment of our work and for the insightful comments and suggestions. Below, we report the statements by Reviewer 1 in italics, followed by our replies.

Comments:

1. How were the other health professionals (571) included in the analysis? Or just in the methodology of the study?

R: We thank the Reviewer for these questions as they allowed us to clarify the point on other health professionals. In addition to nurses and physicians, other health workers – safety inspectors, controllers, administrative personnel, biologists, and researchers – were also invited to participate in the survey. This was done to capture the impact of COVID-19 first wave on these professionals, who were often reassigned as contact tracers during the COVID-19 first wave, and to more accurately account for the regional disparities in the availability of healthcare workers. The results on the entire sample include also the other health professionals. We clarify now this in the “Participants” section at page 4.

2. The authors mention the nurses' resilience and that they have less intention of changing profession or specialization. Resilience is due only to personal factors or also to the employability of nurses compared to doctors, reducing the intention to change profession or specialization.

R: We thank the Reviewer for highlighting this aspect. We do not exclude that differences in employability affect nurses resilience. Yet, our main specification controls for regional fixed effects, which do account for the time invariant labor market characteristics. This means that we produce estimates of the importance of personal characteristics on resilience while controlling for the labor market characteristics. Overall, while the baseline level of resilience might differ between physicians and nurses – in our study nurses report a higher willingness to change profession and specialization than physicians (see Fig.4) – working with positive patients or in a COVID-ward decreases the willingness to change job or specialization only among nurses, showing the resilience of their vocation through these difficult times.

Reviewer #2:

Comments:
1. #P 2 Line 37: after [18] There must be some word, it seems to be missing.

R: We thank the Reviewer for noticing this. We have changed the text as follow:

> "[…] **The study by** [**19**] show**s** that the number of office days is an important determinant of job satisfaction, and that the turnover intentions vary with age among healthcare workers in Bolivia. […]" (p. 2)

2. #P2 Line 46-53: These statements should not be included in the introduction part with the discussion, theses should be moved to the discussion part. As well as line 64 onwards till the end of the introduction part. Introduction part should be written in past tense as well the methodology part.

R: We thank the Reviewer for these suggestions on how to improve the introduction and the organization of the paper. We moved the statements of lines 46-53, and from line 64 onward, in the "Discussion" section, which, in the new version of the manuscript, is separated from the "Results" section, following the suggestion of Reviewer 4. Moreover, we have changed the Introduction and the Methodology part so that now we only use past tenses.

3. #P5 Research design should the written before the heading of "materials and methods"

R: Following this comment and also the comments of the other Reviewers, we revised the structure of the paper. Now, the research design comes immediately after the heading "Methods" and it is followed by the section "Participants."

Reviewer #3:

Dear author, this is an interesting paper about the job satisfaction of healtcare workers after the onset of Covid-19 pandemic. However, the study design doesn't allow to evaluate it's effective impact on those workers, because no data are available about the status of the sample before the pandemic. A longitudinal study could be the best choice to perform the evaluation you intented to do. In addition, to make a stronger evaluation of job satisfaction, you could use a validated tool like The Job Satisfaction Survey (JSS) or others.

R: We thank Reviewer 3 for the positive assessment of our work and for these important remarks which helped us clarifying some key points that did not receive enough attention in the previous version of the paper. We agree with the Reviewer that a longitudinal study would have been the ideal setting to evaluate the change in the level of satisfaction with respect to the period before the pandemic. However, this is not possible since there are no panel of healthcare workers available. Ours is among the first attempt to collect questionnaires from a large sample of

healthcare workers and such has no precedent. The strength of our sample is to cover both physicians and nurses (besides other types of personnel) at the country level, and not at the level of a single hospital. The shortcoming is that it is not possible to compare their status at the moment of the survey, with previous statuses. As far as follow ups are concerned, in respect with the privacy regulations, our survey is completely anonymous and we would not being able to contact the respondents even if we would have liked. We were not able to identify the respondents. Before running our experiment, we tried to contact several survey companies but none of them could rely on a proper panel of physicians and nurses, this is why we resort to our own managed survey.

We still believe our cross-sections approach provides a valid analysis for several reasons. First, the literature identifies two groups of drivers for job satisfaction among healthcare workers: a traditional group made up of personal and contextual factors – which includes socio-economic factors (e.g., gender, age, wages, and workload) and workplace characteristics (e.g. type of hospital, type of employment contract) – and a COVID-19 group (e.g., being exposed to the virus). Considering the first group of drivers, we believe, that most of the personal and contextual factors do not vary substantially over time, especially in a short time period (such as one or two years) – as for the gender, the hours of work, the salary, the quality of the working facility, or the presence of the medical personnel. Our analysis on the impact of these factors on job satisfaction could be considered more constant across time. This is also reassured by the fact that our results are well in line with the findings reported in the literature of interest.

In any case, since we do agree with the Reviewer, the limitations stemming from the lack of a panel analysis are now stressed in the Limitations Section.

Third, participants were explicitly asked to only consider the period from the end of February 2020 to the beginning of June 2020 when answering these questions. In this way, we are able to capture the relationship between the working conditions during the emergency situation (e.g. working overtime) and the level of (rather than the change in) job satisfaction, on top of the standard working conditions (e.g. average hours worked, or contract with work-shifts).

The questions to measure job satisfaction were not conceived by us, but we relied on the validated questions routinely done in the Section C of the Labor Force Survey ("Rilevazione sulle Forze di Lavoro") by the National Institute of Statistics (ISTAT). We now clarify this point in the section "Outcomes and covariates".

Below, we answer the other points raised by Reviewer 3, reporting in italics the Reviewer' statements followed by our replies.

Comments:

1. Many studies performed in italy in the same period have not been cited, even if they could help to better understand the context in which the study has been performed (i.e. De Sio et al).

R: We thank the Reviewer for suggesting the study by De Sio et al. which is now included in the Introduction.

2. English language is quite good even if there are many long sentences and others are quite complex to be understood.

R: We thank the Reviewer for this positive assessment. We have submitted the paper for proofreading at AEJ. To check the certificate on the AJE website it should be used the following verification code: 082F-C6CC-F1B8-586E-F1EP.

3. No response rate has been provided.

R: We agree that no proper explanation for the lack of this information was provided in the previous version of the paper. It is not possible to compute our exact response rate since our survey circulated also through healthcare personnel associations and unions. We can only approximate it. We have now clarified these points in footnote 3 at page 3.

4. The introduction section is too long and, at the end, it deals with results and conclusions that should be put at the end of the paper, not at the beginning.

R: We thank the Reviewer for this comment and for suggesting how to improve the organization of the paper. In light of this comment and those of Reviewer 2 and Reviewer 4, the Introduction has been shortened. In particular, we moved the statements between lines 46-53 and from line 64 onward in the "Discussion" section, which, in the new version of the manuscript, is separated from the "Results" section, following the suggestion of Reviewer 4.

5. The limits of the research have not been disclosed (selection bias etc)

R: We now have included a Limitations section.

6. The authors state that they focused on the respondents from northern Italy, but they didn't indicate how many respondents came from there and how many from other parts of the country

R: We report the distribution of the responses by region and profession in Figure 2. However, we agree that from this figure it is not clear what is the exact number of respondents within the northern area and each region. In order to clarify this information to the reader we added the footnote 2.

7. Many question of the survey have not been justified in relation to the aims of the study (i.e. homes dimensions etc).

R: We agree that in the previous version of the manuscript it was not obvious why we had inserted some questions. Therefore, we have reviewed the "Outcomes and covariates" Section accordingly.

Reviewer #4:

Comments:

1. Introduction: "with more than 7,000 respondents (about 2,500 physicians and 4,500 nurses) conducted". Please designate the exact numbers of participants.

R: We thank the Reviewer for this comment. We now report the exact figures: 7,681 respondents (about 2,549 physicians and 4,561 nurses) .

2. Introduction section is too long. It is recommended the authors summarize the Introduction section (in 1000-1500 words) representing the importance of the study and how the study can fill the literature gap. Many paragraphs-describing the similar articles in other countries- can be discussed in the Discussion section.

R: We thank the Reviewer for this comment and for suggesting how to improve the organization of the paper. In light of this comment and the point raised by Reviewer 2 and Reviewer 3, we shortened the Introduction which is now between the suggested word limits.

3. Please follow the guidelines of scientific writing.

R: Following the PLOS ONE guidelines on how to edit the manuscript (here: https://plos.org/resource/how-to-edit-your-work/), we also have submitted the paper for proofreading at AEJ. To check the certificate on the AJE website it should be used the following verification code: 082F-C6CC-F1B8-586E-F1EP.

4. Please cite the following Covid-19 related article in the Introduction section:
- https://www.ncbi.nlm.nih.gov/pmc/articles/PMC8184167/

R: We added the suggested reference in the Introduction.

5. Introduction: It may not be necessary to describe the first Covid-19 wave in Italy in details. It is suggested the authors summarize this section in a paragraph.

R: We shortened the description of the first Covid-19 wave in Italy to a paragraph and we included it in the Introduction.

6. Figs. 1 and 3 can be submitted as supplementary materials.

R: We moved Fig. 1 and 3 in the supplementary materials.

7. Please follow the Journal guideline to prepare the manuscript. For example, the Results and Discussion sections must be presented separately. It is recommended the author refer to the PLOS ONE guideline for authors.

R: We have followed the suggestion of the Reviewer so that in the new version of the manuscript the “Discussion” section is separated from the “Results” section.

8. Please add a table summarizing the basic characteristics of the participants.

R: We show the summary statistics with the observable characteristics of our participants in Tables 4,5,6.

9. Many figures have been submitted. It is recommended the authors merge Figs. 4-12 to three figures. Figure 13 can be explained in the text and be omitted. Figure 2 can be submitted as a supplementary material.

R: We have decreased the number of Figures. Fig. 2 has been moved to the supplementary material, Fig. 13 does no longer appear and we reorganized Fig. 4-12. However, we could not group them to three as they refer to different issues and are discussed in different parts of the paper. Specifically, we merged together figures 6, 7 and 8, and figure 9 with figure 10. We have changed the text to accomodate these variations.
